# Identification of ocular refraction based on deep learning algorithm as a novel retinoscopy method

**DOI:** 10.1186/s12938-022-01057-9

**Published:** 2022-12-17

**Authors:** Haohan Zou, Shenda Shi, Xiaoyan Yang, Jiaonan Ma, Qian Fan, Xuan Chen, Yibing Wang, Mingdong Zhang, Jiaxin Song, Yanglin Jiang, Lihua Li, Xin He, Vishal Jhanji, Shengjin Wang, Meina Song, Yan Wang

**Affiliations:** 1 Clinical College of Ophthalmology, Tianjin Medical University, Tianjin, China; 2 Tianjin Key Lab of Ophthalmology and Visual Science, Tianjin Eye Institute, Tianjin Eye Hospital, Nankai University Affiliated Eye Hospital, 4 Gansu Road, He-Ping District, Tianjin, 300020 China; 3 School of Computer Science, School of National Pilot Software Engineering, Beijing University of Posts and Telecommunications, 10 Xitucheng Road, Hai-Dian District, Beijing, 100876 China; 4 HuaHui Jian AI Tech Ltd., Tianjin, China; 5 Tianjin Eye Hospital Optometric Center, Tianjin, China; 6 UPMC Eye Center, University of Pittsburgh School of Medicine, Pittsburgh, PA USA; 7 Department of Electronic Engineering, Tsinghua University, Beijing, China; 8 Nankai University Eye Institute, Nankai University, Tianjin, China

**Keywords:** Deep learning, Fusion model, Ocular refraction, Retinal fundus photographs, Cycloplegic refraction

## Abstract

**Background:**

The evaluation of refraction is indispensable in ophthalmic clinics, generally requiring a refractor or retinoscopy under cycloplegia. Retinal fundus photographs (RFPs) supply a wealth of information related to the human eye and might provide a promising approach that is more convenient and objective. Here, we aimed to develop and validate a fusion model-based deep learning system (FMDLS) to identify ocular refraction via RFPs and compare with the cycloplegic refraction. In this population-based comparative study, we retrospectively collected 11,973 RFPs from May 1, 2020 to November 20, 2021. The performance of the regression models for sphere and cylinder was evaluated using mean absolute error (MAE). The accuracy, sensitivity, specificity, area under the receiver operating characteristic curve, and F1-score were used to evaluate the classification model of the cylinder axis.

**Results:**

Overall, 7873 RFPs were retained for analysis. For sphere and cylinder, the MAE values between the FMDLS and cycloplegic refraction were 0.50 D and 0.31 D, representing an increase of 29.41% and 26.67%, respectively, when compared with the single models. The correlation coefficients (*r*) were 0.949 and 0.807, respectively. For axis analysis, the accuracy, specificity, sensitivity, and area under the curve value of the classification model were 0.89, 0.941, 0.882, and 0.814, respectively, and the F1-score was 0.88.

**Conclusions:**

The FMDLS successfully identified the ocular refraction in sphere, cylinder, and axis, and showed good agreement with the cycloplegic refraction. The RFPs can provide not only comprehensive fundus information but also the refractive state of the eye, highlighting their potential clinical value.

**Supplementary Information:**

The online version contains supplementary material available at 10.1186/s12938-022-01057-9.

## Background

Refractive errors are the most common ocular disorders and the second leading cause of blindness [1–3]. Recently, the distribution of refractive errors worldwide has shifted towards myopia. Myopia has become an epidemic-like public health issue due to its soaring incidence and prevalence, and potentially long-term associations with sight-threatening ocular complications [4]. Hence, precise measurement and assessment of refraction are essential for evaluating the degree of ametropia and providing appropriate eye care. Clinical subjective refraction under cycloplegia is a routine technique for determining refractive errors. However, the procedure is laborious, time consuming, and can sometimes result in blurred vision, photophobia, and the perception of glare due to pupil dilation [5, 6]. ​Additionally, it is inconvenient and can be challenging for disabled or paediatric patients, especially in resource-limited settings. Even with the advent of autorefractors, the results of refraction measurement remain unsatisfactory because of the accommodation [7]. In addition to overestimating the prevalence and severity of myopia, these devices could affect preventive and corrective strategies for myopia. Despite the traditional subjective refraction as the gold standard, such procedures are commonly marred by long measurements with low repeatability. Thus, the future trend is to overcome the traditional manual method and provide faster measurements with lower variability. Unfortunately, data concerning refraction and its association with retinal fundus photographs (RFPs) are lacking. Therefore, a more effective method should be developed to improve detection, documentation, and prediction of refraction.

Fundus photography can objectively reflect retinal morphology and is commonly applied in clinical practice. Changes in myopia cause distortion of the retinal image and deterioration of visual quality. The typical features of retinal morphology in myopes are parapapillary atrophy, tessellation, and changes in macular regions or arterial trajectories. These changes are more pronounced in patients with high and pathological myopia [8–12]. In addition to these visible structures, fundus image intensities represent the amount of reflected light, which provide information on the complete state of the eye. Whether this information informs on ocular refraction and explains image distortions caused by astigmatism remains elusive.

Artificial intelligence (AI) has been extensively applied in the classification and prediction of medical data [13–15]. Most of these studies were retrospective in nature. However, external validation and algorithm testing in the prospective trials are indispensable for clinical transformation. In this direction, some clinical trials have developed reliable machine learning or deep learning (DL) tools, including AI-assisted decision-making for refractive surgery [16], DL-based prediction of breast cancer chemotherapy [17], and computer-aided diagnosis of gastric cancer risk [18]. The broader capacity of AI was applied to extract regions of interest (ROI) that physicians typically cannot recognize from images alone, thereby providing greater clinical insights and findings [19], such as the identification of Alzheimer’s disease and monitoring of cardiovascular diseases from fundus images [20, 21]. Furthermore, several studies had reported the performance of AI in determining refractive errors based on various types of data [22–26]. However, owing to differences in training data and target values, most models output spherical equivalent (SE), which is not suitable for clinical practice. More importantly, these studies did not determine the cylinder axis.

Therefore, here, we developed a novel fusion model-based deep learning system (FMDLS) to effectively and accurately identify ocular refraction from RFPs and compared it to the cycloplegic refraction in sphere, cylinder, and axis.

## Results

### Baseline characteristics

Overall, 11,973 images (6086 patients) were collected, 7873 images (3954 patients) of which were processed and retained. A total of 7086 images were eventually randomly selected to construct the regression model (RM) and classification model (CM) for sphere and cylinder, respectively, whereas the remaining 787 images were used for testing. Among the total images, 2028 were used for the CM of the cylinder axis as the uneven axial distribution in the crowd. Patients’ age ranged from 6 to 40 years, with a mean (standard deviation, SD) of 18.5 (7.3) years. The mean sphere was − 3.82 D (2.05 D) (range: − 0.25 to − 8.00 D) and the mean cylinder was − 0.82 D (0.61 D) (range: 0 to − 2.75 D). We categorized the data to ensure that images acquired from the same patient were not split across the training and validation sets (Table 1).

**Table 1:** Summary of the training, validation, and test sets

	Training set	Validation set	Test set
No. of patients	2769	791	394
Sex, (M/F)	1548/1221	351/440	173/221
Age (y), mean (SD)	18.35 (6.50)	18.72 (7.34)	18.94 (7.22)
RM (No. of images)	5511	1575	787
CM (No. of images)	5511	1575	787
A-CM^a^ (No. of images)	1420	406	202
Sphere, mean (SD)	− 3.77 (2.04)	− 3.95 (2.05)	− 3.95 (2.09)
Cylinder, mean (SD)	− 0.82 (0.61)	− 0.81 (0.60)	− 0.83 (0.63)
Axis (W/A/O)	2920/1543/1048	882/504/189	519/204/64
SE, mean (SD)	− 4.18 (2.11)	− 4.36 (2.12)	− 4.17 (2.12)
High myopia	36.9%	39.6%	36.2%
Moderate myopia	28.7%	29.1%	28.7%
Mild myopia	34.4%	31.3%	35.1%
Intraocular pressure (mmHg)	16.1 (2.01)	15.9 (2.16)	16.4 (1.78)
Uncorrected distance visual acuity (LogMAR)	0.68 (0.25)	0.69 (0.21)	0.69 (0.22)
Centre corneal thickness	551.57 (30.93)	555.17 (22.18)	547. 28 (22.61)
K1	42.41 (1.25)	42.35 (1.33)	42.41 (1.31)
K2	43.75 (1.41)	43.99 (1.36)	43.96 (1.43)

*M* male, *F* female, *SD* standard deviation, *RM* regression model, *CM* classification model, *A* axis, *W* with-the-rule, *A* against-the-rule, *O* oblique, *SE* spherical equivalent, *LogMAR* logarithm of the minimum angle of resolution, *K* keratometry

^a^Only classification model

### Performance of the FMDLS in test set

**Fig. 1:**
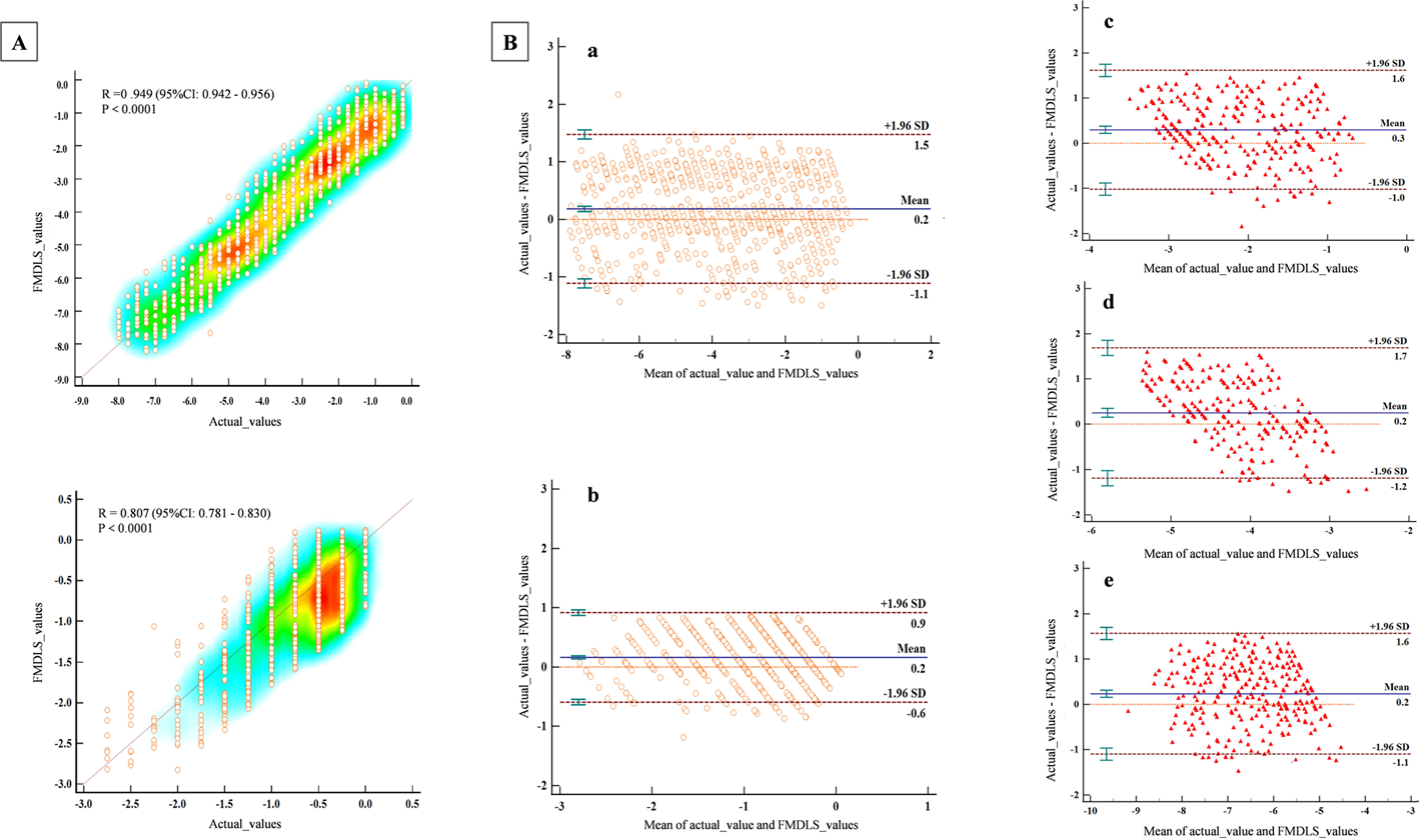
Relationship of the FMDLS and actual values. **A** (upper left and bottom left): the overall distribution of the FMDLS and actual values; the *Y*-axis represents the FMDLS values and *X*-axis represents the actual values. Upper is sphere. Bottom is cylinder. **B** (middle and right pictures): the Bland–Altman plot of the FMDLS and actual values in the test set; the *Y*-axis represents the difference between the values, and the *X*-axis represents the average of the two values. Pictures **a** and **b** are the performance of FMDLS in the sphere and cylinder, respectively; **c** is mild myopia; **d** is moderate myopia; and **e** represents high myopia

According to the results of the confusion matrix, we compared the performance of FMDLS with and without age as the eigenvector. The performance of each model (RM and CM) and the FMDLS for the test set are listed in Table 2. For sphere and cylinder, the mean absolute error (MAE) of the RMs were 0.66 D and 0.38 D, respectively. The area under the curve (AUC) values of the CMs were 0.863 [95% confidence interval (CI) 0.839–0.887] and 0.834 (95% CI 0.808–0.860), respectively, with the AUC values of 0.8–0.9 indicating excellent performance [27]. The accuracy, specificity, sensitivity, and F1-score are shown in Table 2. For the FMDLS, the MAEs of sphere and cylinder were 0.50 D and 0.31 D, representing 29.41% and 26.67% increases, respectively, with respect to those for the RM. The overall distributions of the FMDLS and actual values were almost in a good agreement with those shown in the scatter diagram in Fig. 1A. The Pearson’s correlation coefficient (*r*) values were 0.949 (95% CI 0.942–0.956) and 0.807 (95% CI 0.781–0.830), respectively. Figure 1B shows the Bland–Altman plot comparing the FMDLS and actual values in the test set. For the classification of the cylinder axis, the AUC value was 0.814 (95% CI 0.708–0.902).

**Table 2:** Performance of single models and the FMDLS

	RM	CM					FMDLS		
	MAE	Accuracy (95% CI)	Specificity (95% CI)	Sensitivity (95% CI)	AUC (95% CI)	F1-score	MAE	*r*	Performance improvement
Sphere^a^	0.86	0.790 (0.751–0.842)	0.991 (0.974–0.998)	0.795 (0.745–0.839)	0.798 (0.748–0.842)	0.775	0.63	0.815	27.10%
Sphere^b^	0.66	0.850 (0.825–0.875)	0.996 (0.99–0.998)	0.859 (0.835–0.883)	0.863 (0.839–0.887)	0.828	0.50	0.949	29.41%
Cylinder	0.38	0.860 (0.836–0.884)	0.989 (0.982–0.996)	0.861 (0.837–0.885)	0.834 (0.808–0.860)	0.863	0.31	0.807	26.67%
Axis	–	0.890 (0.816–0.964)	0.941 (0.849–0.981)	0.882 (0.776–0.944)	0.814 (0.708–0.902)	0.880	–	–	–

*RM* regression model, *CM* classification model, *FMDLS* fusion model-based deep learning system, *MAE* mean absolute error, *AUC* area under the curve

^a^Model without age as an eigenvector

^b^Model with age as an eigenvector

### Model visualization

**Fig. 2:**
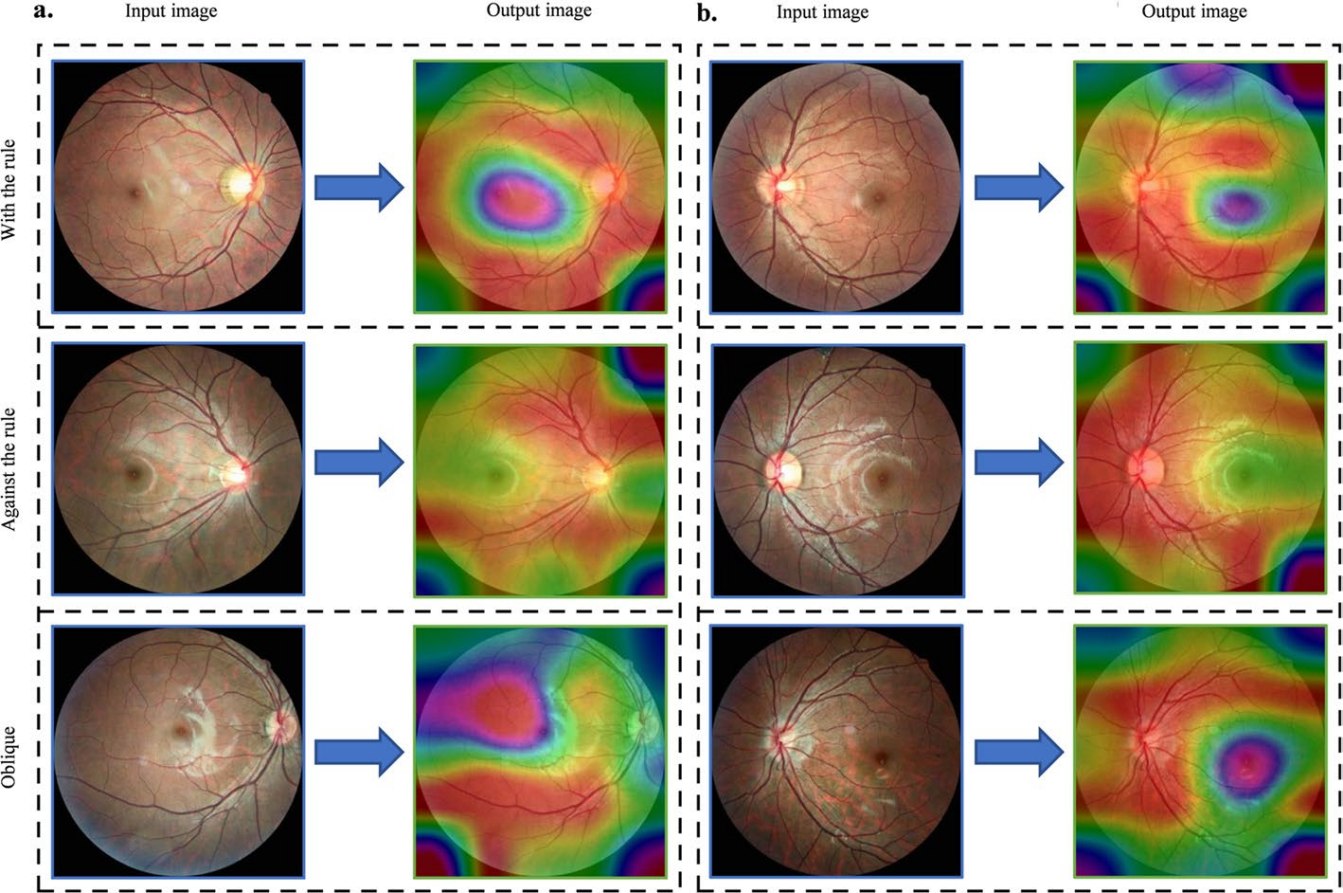
Attention maps of the eyes with three categories of astigmatism detected using FMDLS. **a** Original image and visualization of the right eye; **b** original image and visualization of the left eye

To better visualize how the FMDLS was able to detect the cylinder axis from the RFPs directly, the attention maps were superimposed on the convolutional visualization layer generated to understand the contributions of the ROIs (Fig. 2). The retinal vascular regions were highlighted in these maps, and as a fundamental feature appeared in all images. Additionally, the macular areas, as another ROI, existed only in the with-the-rule (WTR) group and the oblique group. These observations were found in nearly all images.

## Discussion

In this study, we developed and applied a novel FMDLS to identify the ocular refraction and compared it to the clinical gold standard. To our knowledge, this was the first FMDLS simultaneously analysing both sphere, cylinder (mean difference: 0.5 D and 0.31 D, respectively) and cylinder axis (AUC value: 0.814). The results derived from this system showed a strong correlation with clinical cycloplegic refraction (*r* = 0.949 and *r* = 0.807, *P* < 0.0001). Importantly, the study proved that the FMDLS was promising when considering all metrics (including sphere, cylinder, and axis). We further evaluated the performance of the different subgroups of refraction and found that the FMDLS could identify different refraction through common clinical retinal images with a consistent performance. It was proven that the FMDLS had the potential of owning a beneficial effect on refractive assessment due to its ability to represent the state of the human eye objectively and comprehensively.

As cycloplegic refraction was inconvenient and limited in large-scale screening procedures [28], non-cycloplegic refractive tests had been employed more frequently in emerging studies to determine ametropia. Simultaneously, AI-based methods to predict refractive errors via ocular images had been promising new hotspots of research [25]. In particular, the consensus among these approaches was to allow algorithms to learn predictive features directly from a large number of labelled images without explicitly specifying rules or features [29]. However, the output of these algorithms only included the SE (SE = sphere + 1/2 cylinder) and could not reflect the complete status of patients [24, 30]. The system in the current study had overcome this shortage and obtained the considerable results. Several studies had identified and segmented the retinal visible structures of the myopes based on AI algorithms, including the optical disc, fovea, and tessellations [31, 32]. In fact, shifting myopia degrees could lead to these structural changes, making it possible for automatic myopia identification and detection, as DL algorithms could easily detect structural changes from fundus images. Moreover, these images also contain valuable and inconspicuous information, such as the light that reflected from the retina, lens, and cornea. The comprehensive information available from the data might be leveraged by the new FMDLS. Notably, the current FMDLS was more objective and practical and reached better predictive performance than cycloplegic refraction, making it appropriate for clinical usage.

Furthermore, we extracted the ROIs during model training and obtained the sphere and cylinder based on data from the entire retina, embracing the optical disc tilt, atrophy, and fovea morphology. Vascular regions were especially highlighted as a previously unnoticed feature. Further analysis of the cylinder axis using attention maps revealed informative features and locations. Interestingly, consistent focus on the vessels in the attention maps could indicate the axial results, and this had not been reported in previous studies. Different categories of astigmatism were also identified in different regions on the maps. The WTR astigmatism was usually focused on areas parallel to the retinal blood vessels, whereas against-the-rule (ATR) astigmatism was focused on areas perpendicular to the vessels. Almost all areas of the optic disc could be observed across the three categories, although the macular region could not be observed in cases of ATR astigmatism. Oblique astigmatism did not seem to follow a specific distribution in the attention map and was mainly focused on the macular area.

Astigmatism was mainly from the differential amplification of major corneal meridians, but astigmatism assessment based on cornea alone was inaccurate [23]. When light passed through different meridians, the differences in refractive power could induce blurred images, causing retinal image distortion along the axis [6, 33]. The attention maps in the study highlighted this possibility and indicated a correlation between the ROI and anatomy. A previous study reported that astigmatism could induce changes in the thickness of the retinal nerve fibre and optic nerve head parameters during optical coherence tomography [34]. Chameen et al. [10] found that the distributions of the disc tilt axis and corneal curvature were similar, and astigmatism exhibited a strong relationship with retinal anatomy and suggested the same embryological origin. The findings of the current studies laid a foundation for understanding how the model identified this information. Although they did not establish causation, these maps might explain the image distortion caused by astigmatism and could help generate unbiased hypotheses for further study of the cylinder axis [35].

Measuring refraction without accommodation had been the standard for detecting myopia [36]. To achieve this, cycloplegic agents needed to be administered, especially in paediatric patients with a wide range of accommodations. The prevalence and severity of myopia were overestimated when cycloplegic agents were withheld [28]. Despite differences in the use of cycloplegic agents, measurement methods, age ranges of participants, and refractive status among studies, the reported mean difference between non-cycloplegic and cycloplegic refractive errors ranged from 0.62 D to 1.23 D, with inter-method differences significantly decreasing with age [37]. Compared with cycloplegic refraction, the ocular refraction analysed using our system performed with clinically acceptable accuracy and largely corrected the overestimation of myopic shift. More particularly, it was helpful for evaluating different degrees of astigmatism.

Our system achieved a medical application of AI; the results demonstrated that personalized modelling with a convolutional neural network (CNN) and CNN-based transfer learning was an improved estimation approach that could be used across diverse patient subgroups. Age was used as a contributing feature to improve performance. The system was developed using the clinical gold standard as the target to separately identify refractive errors in sphere, cylinder, and axis, and the feature extractors using the XGBoost algorithm reduced model variance, increased its robustness, and prevented overfitting of the class-unbalanced population data. We introduced a voting mechanism for validation, which allowed us to combine the single models while increasing accuracy and reducing bias. Indeed, RFPs were collected from patients at different time points; hence, the lighting and background of the images were not uniform, indicating the richness and diversity of our datasets. Also, it should be cleared that the algorithm mainly focuses on the landmarks in fundus images to predict the refraction. Naturally, testing on invisible fundus images with disease artefacts or lens artefacts may result in increased error compared to the ground truth. As fundus photography is used worldwide, and portable and affordable cameras are becoming more common and popular, this system is expected to have greater advantages for large-scale surveys. In short [38], the present approach enables integrated observation of retinal conditions and simultaneous assessment of refractive errors.

This study had several limitations. First, the imbalance of high myopia and astigmatism in the dataset might have affected the overall performance, although we included the relative outliers and minority classes with larger weights in the training set to address this problem. Second, data were collected from the same type of fundus camera, and the homogeneity of images was much higher than in other studies and situations. The absence of images from other sources limits the generalizability of the system. Finally, we excluded patients diagnosed with other ocular diseases, and changes in the fundus were only due to refractive errors. Future studies should utilize a larger multi-centre dataset and additional clinical results to determine the clinical applicability.

## Conclusions

In this study, we developed an FMDLS as a novel retinoscopy method to identify the ocular refraction, and the results were generally consistent with cycloplegic refraction measurement. This system was capable of assessing ocular refraction reliably and directly, avoiding time-consuming cycloplegic process. Importantly, the attention maps generated from the system might provide new perspectives to explain the image distortion caused by myopic astigmatism and help determine imaging biomarkers for diagnosing refractive errors. These findings also highlight the potential values of AI-based model to provide detailed information on both retinal changes and refraction states simultaneously. In the future, combining FMDLS with smartphones might further enable patients to self-monitor refraction changes and might have potentially significant implications for eye care worldwide, especially in areas with limited healthcare resources.

## Methods

### Ethics statement

This study was registered in the Chinese Clinical Trial Register (ChiCTR2100049885), approved by the Ethics Committee of Tianjin Eye Hospital, and conducted in accordance with the tenets of the Declaration of Helsinki. The ethical committee waived the requirement for informed consent owing to the retrospective study design and the use of anonymized RFPs. This study followed the Standards for Reporting of Diagnostic Accuracy Study-AI (STARD-AI) reporting guidelines [39].

### Data collection

The dataset was retrospectively collected from medical records at Tianjin Eye Hospital of Nankai University from May 1, 2020, to November 20, 2021, and analysed in December 2021. Relevant demographic information included sex and age; ocular parameters included uncorrected visual acuity, intraocular pressure (Topcon Inc., Tokyo, Japan), corneal morphology from Pentacam HR (Oculus Inc., Wetzlar, Germany), and fundus images captured by CR-2 AF non-mydriatic retinal camera (Canon Inc., Tokyo, Japan). We collected images with refractive errors alone and excluded patients with any other ocular diseases, such as corneal diseases, cataract, glaucoma, retinal disease, and a history of intraocular surgery. The values and parameters of both eyes were used in the main statistical analyses. Clinical subjective refraction was measured after cycloplegia, with sphere ranging from 0.75 D to − 10.00 D and cylinder ranging from 0 D to − 6.00 D. According to the SE refraction, the subgroups were identified as mild myopia (− 3.0 D ≤ SE ≤ − 0.50 D), moderate myopia (− 5.00 D < SE < − 3.00 D), and high myopia (SE ≤ − 5.00 D) [40]. All measurements were performed by three optometrists with more than 10 years of experience, and there were no significant differences in the consistency of assessments. Overall, 11,973 images taken in 6086 patients at different time points were collected without pupil dilation. All images were acquired with a 45° field-of-view centred on the fovea.

The images were filtered according to the following criteria. (1) Images with complete fundus information were retained, including anatomical structures, such as optic disc, macula, and vessels. (2) Images with extremely low resolution, significant artefacts, or blurring were discarded. (3) Size and resolution were normalized for all images with the same magnification ratio and form. Furthermore, each image was labelled with the corresponding cycloplegic refraction, and the refractive status of each image was determined using the sphere, cylinder, and axis. The cleaned images were retained and divided into the training, validation, and test sets at a ratio of 7:2:1. The process of data collection is shown in Additional file 1.

### Data pre-processing and augmentation

**Fig. 3:**
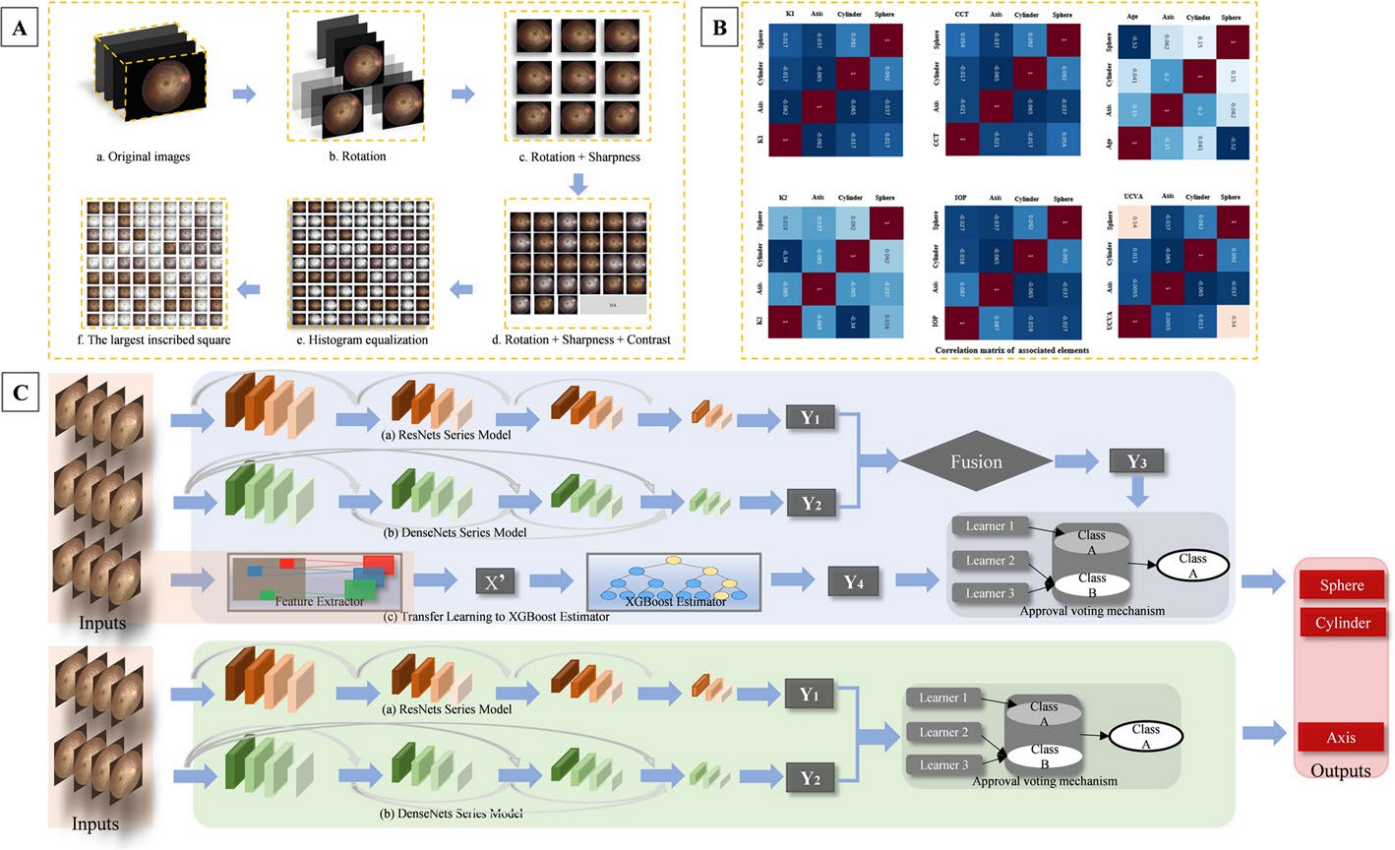
Diagram of the system construction. **A** Image pre-processing and augmentation; **a** original RFPs; **b** the largest inscribed circle and rotation; **c** rotation and sharpness; **d** contrast-limited adaptive histogram equalization was used to improve colour and spatial contrast between the structures and the background retina for RFPs; **e** histogram equalization processing; **f** the largest inscribed square. **B** Confusion matrix between target values and eigenvectors. The colours in the figure indicate the strength of the correlation. **C** Architecture of the FMDLS proposed in this study. (Upper picture) the pipeline for sphere and cylinder, **a** and **b** were networks for two different models, **c** was the classification model; (Bottom picture) the pipeline for axis, **a** and **b** were two different classification models

To retain as much practical information as possible in all images, the Hough transform was used to locate the optimal image boundary, determine the centre and radius of the standard circle, and construct the largest inscribed circle and square. Contrast-limited adaptive histogram equalization was used to extract the red and green channels from an image to highlight the vascular structure and enhance contrast. We removed the proportion of invalid pixels to maintain the fundus as the largest inscribed circle within the area (Fig. 3A, b), followed by the largest inscribed square (Fig. 3A, f). Finally, the image was converted to a resolution of 512 × 512 pixels.

Data augmentation was performed during pre-processing: (1) random rotation was performed between − 30° and + 30° based on the original angle; (2) the sharpness was randomly adjusted to 0.5×, 1×, or 2× the original image; (3) the contrast was automatically set with a probability of *p* = 0.5; (4) the histogram of the image was randomly equalized with a probability of *p* = 0.5 (Fig. 3A, b–e). Data augmentation methods are presented in Additional file 2.

### Construction of the FMDLS

Before constructing the system, the recorded parameters were filtered to determine which could be used as the eigenvectors (Fig. 3B). We further applied discrete variables scattered in the space with units of 0.25 D as labels, and sphere and cylinder as the target to ensure the output were clinically appropriate. Two different algorithms were adopted to construct the RM and CM. The specially designed voting mechanism was applied in the bagging stage to enhance the accuracy and overall generalizability of the models.

Considering the severe imbalance in the distribution of the axis caused by the population, we divided the data into the following three categories based on the type of astigmatism: WTR, ATR, and oblique (Fig. 3C).

### Regression models

The training data were utilized to construct the RMs for sphere and cylinder. The mean and SD of the red, green, and blue channels of the images were calculated and normalized based on the results. We then input the normalized matrix into the pre-trained neural network. As age was easy to obtain and had an obvious correlation with sphere, we attempted to normalize age into an independent eigenvector as the input of the extreme gradient boosting (XGBoost) algorithm (Fig. 3C, c) to train and adjust the parameters. The MAE was selected as the loss function of XGBoost during the stage. The normalization method remained unchanged during the training and testing phases. Residual Network (ResNet-34) was used as the backbone network, revising the output dimension of the final fully connected layer to one. Without loading pre-training parameters, we used the MAE as the loss function and trained from scratch.

### Classification models

The sphere and cylinder were regarded as discrete variables, and 0.25 D was used as the minimum distance of the variable interval when constructing the CMs. The data conforming to the population distribution were selected to alleviate extreme imbalances in categories and avoid the influence of outliers on the construction of the CMs. ResNet-34 (Fig. 3C, a) and Dense Convolutional Network (DenseNet-121) (Fig. 3C, b) were applied to classify the sphere and cylinder, wherein the fully connected layer units were modified to 45 and 18, separately. These models used pre-trained model weights and were fine-tuned during training. Focal loss was used as a loss function to train relative outliers and minority classes with larger weights to alleviate the category imbalance [41]. For cylinder axis, three categories (WTR, ATR, and oblique) were divided based on the clinical data, and categorical differences were reduced by down-sampling.

### Fusion model

A specially designed voting mechanism was applied to build the fusion model during the bagging stage.

$$\frac{{\left( {{\text{MR}}_{{{\text{reg}}}} - \frac{{\sum {\text{GT}}_{{{\text{reg}}}} }}{{n_{{{\text{reg}}}} }}} \right){*}w_{{{\text{reg}}}} + \left( {{\text{MR}}_{{{\text{cls}}}} - \frac{{\sum {\text{GT}}_{{{\text{cls}}}} }}{{n_{{{\text{cls}}}} }}} \right){*}w_{{{\text{cls}}}} }}{2} + \frac{{\sum {\text{GT}}_{{{\text{all}}}} }}{{n_{{{\text{all}}}} }}.$$

In the equation, MR denoted the model prediction value, GT the ground-truth value, *n* the number of samples, and *w* the weight of a specific model. Subscripts represented regression (reg), classification (cls), and all collected datasets (all). The fusion model was obtained via calculating the voting distance and the crowd centre. The left part of the equation, the voting distance was generated by averaging the distances of each model, which were calculated by subtracting the centres of training samples from the model predictions. The right part of the equation, the actual centres were calculated by all the samples. Finally, the new FMDLS was constructed using these algorithms.

### Comparison and evaluation of the FMDLS versus cycloplegic refraction

The performance of the RMs was calculated using the MAE between the prediction and the actual values. The MAE measured the forecast accuracy by averaging the absolute values of the residuals; it provided the average value of the error and expressed in the same units as the original response variable. We also calculated other metrics (accuracy, sensitivity, specificity, AUC value with its 95% confidence interval [CI], and F1-score) to assess the performance of the CM.

### Statistical analysis

All analyses were performed using MedCalc, version 19.6.3 (MedCalc Software, Ostend, Belgium; http://www.medcalc.org). Continuous demographic variables are expressed as mean ± SD, and normality was assessed using the Kolmogorov–Smirnov test. The Pearson’s correlation coefficient (*r*) was used to show the strength of correlations. Bland–Altman plots were used to analyse the agreement between the FMDLS and actual values in different groups. The agreement was quantified by measuring whether 95% of the data points were within 2 SDs of the mean difference. Zero difference between the FMDLS and actual values indicated an ideal agreement [42].

## Supplementary Information

**Additional file 1.** Flowchart of the current study.

**Additional file 2.** The methods of data pre-processing and augmentation.

## Data Availability

Data are available from the corresponding author on reasonable request.

## Abbreviations

AI: Artificial intelligence
AUC: Area under the curve
ATR: Against-the-rule
CI: Confidence interval
Cls: Classification
CM: Classification model
CNN: Convolutional neural networks
DenseNet: Dense convolutional network
F: Female
FMDLS: Fusion model-based deep learning system
LogMAR: Logarithm of the minimum angle of resolution
M: Male
MAE: Mean absolute error
*r*: Correlation coefficient
Reg: Regression
ResNet: Residual network
RFP: Retinal fundus photograph
RM: Regression model
ROI: Regions of interest
SD: Standard deviation
SE: Spherical equivalent
STARD-AI: Standards for reporting of diagnostic accuracy study-AI
WTR: With-the-rule
