# Supplementary figures and images for: Identification of ocular refraction based on deep learning algorithm as a novel retinoscopy method
Source: Biomed Eng Online. 2022 Dec 17;21:87. doi: 10.1186/s12938-022-01057-9 (PMC9758840; doi:10.1186/s12938-022-01057-9)

Flowchart of the current study.

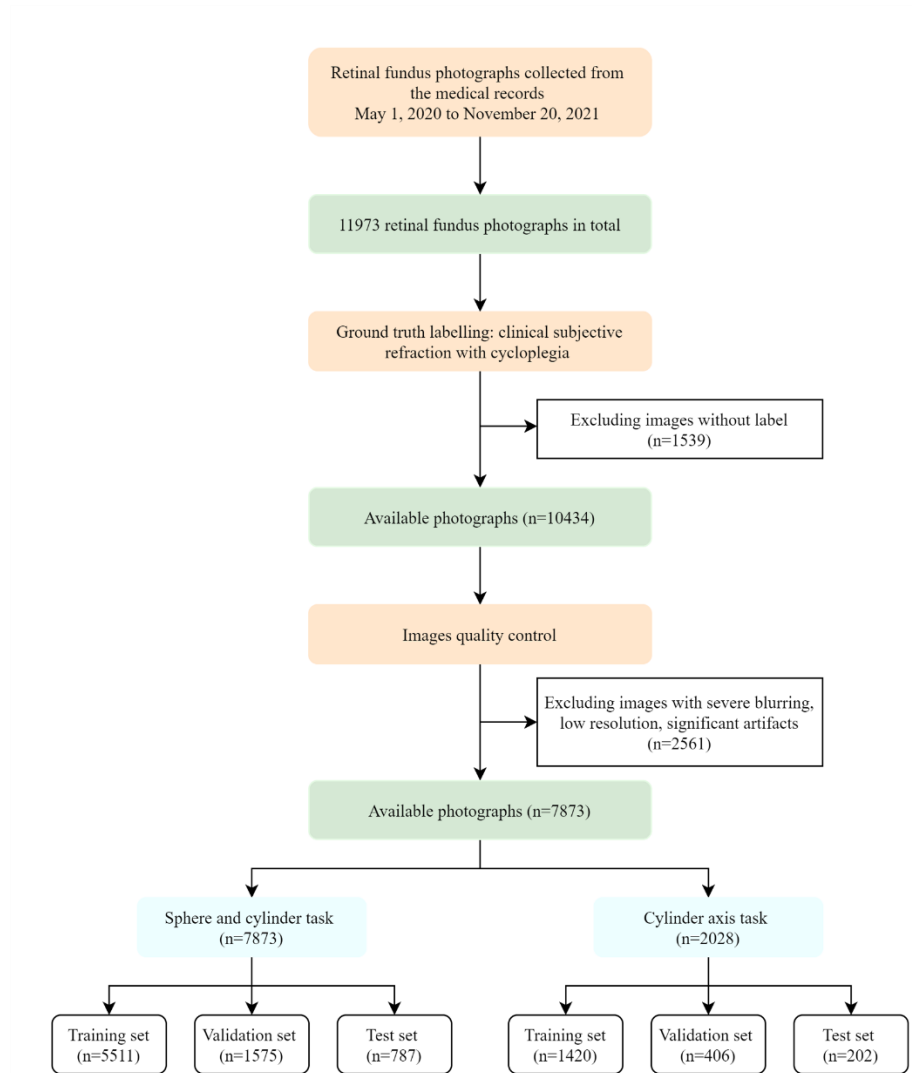

Supplement: Supplementary file 1 — Additional file 1. Flowchart of the current study. [file 12938_2022_1057_MOESM1_ESM.pdf]
