# Supplementary material for: Identification of ocular refraction based on deep learning algorithm as a novel retinoscopy method
Source: Biomed Eng Online. 2022 Dec 17;21:87. doi: 10.1186/s12938-022-01057-9 (PMC9758840; doi:10.1186/s12938-022-01057-9)

Data augmentation methods based on color space transformation and geometric transformation can eliminate the differences between the test set and the training set. In this study, we employed (a) Original fundus images. (b) The largest inscribed circle is processed to retain as much valid information as possible and rotate the images. (c) Rotate and sharpen images. (d) Contrast limited adaptive histogram equalization was used to improve color and spatial contrast between structures and the background for the retina with significant contrast differences. (e) Histogram equalization processing. (f) The largest inscribed square. In order to show the processed characteristics in detail, we selected the original picture and each step of the process. To show the details, we supplement (e) and (f) separately

(e). Histogram equalization processing

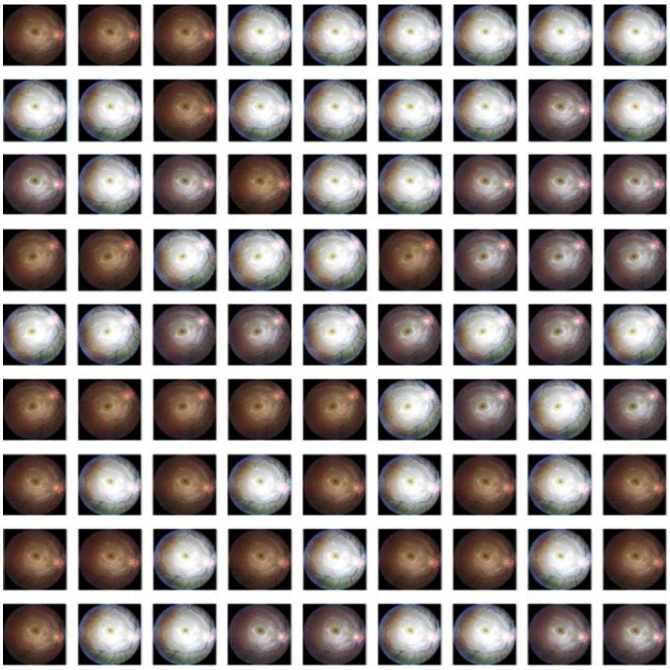

(f). The largest inscribed squares were retained.

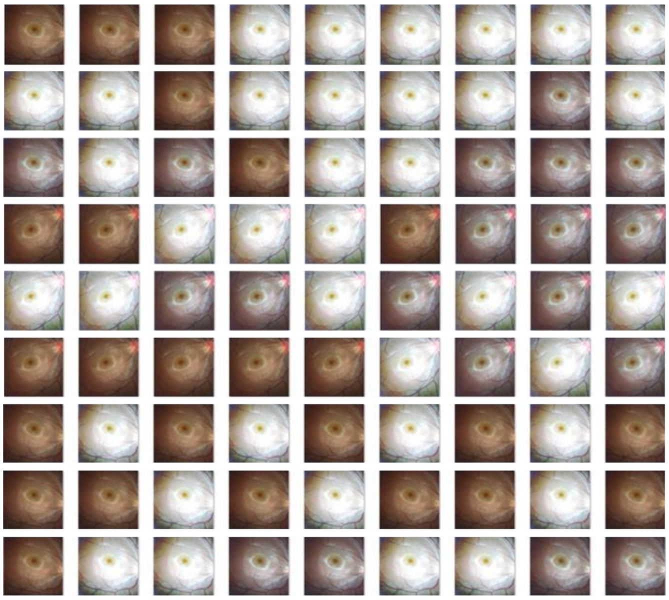

Supplement: Supplementary file 2 — Additional file 2. The methods of data pre-processing and augmentation. [file 12938_2022_1057_MOESM2_ESM.pdf]
